# Characteristics of US Counties With High Opioid Overdose Mortality and Low Capacity to Deliver Medications for Opioid Use Disorder

**DOI:** 10.1001/jamanetworkopen.2019.6373

**Published:** 2019-06-28

**Authors:** Rebecca L. Haffajee, Lewei Allison Lin, Amy S. B. Bohnert, Jason E. Goldstick

**Affiliations:** 1 Department of Health Management and Policy, University of Michigan School of Public Health, Ann Arbor; 2 Injury Prevention Center, Department of Emergency Medicine, University of Michigan Medical School, Ann Arbor; 3 Department of Psychiatry, University of Michigan Medical School, Ann Arbor; 4 Veterans Affairs Center for Clinical Management Research, Veterans Affairs Ann Arbor Healthcare System, Ann Arbor, Michigan

## Abstract

**Importance:**

Opioid overdose deaths in the United States continue to increase, reflecting a growing need to treat those with opioid use disorder (OUD). Little is known about counties with high rates of opioid overdose mortality but low availability of OUD treatment.

**Objective:**

To identify characteristics of US counties with persistently high rates of opioid overdose mortality and low capacity to deliver OUD medications.

**Design, Setting, and Participants:**

In this cross-sectional study of data from 3142 US counties from January 1, 2015, to December 31, 2017, rates of opioid overdose mortality were compared with availability in 2017 of OUD medication providers (24 851 buprenorphine-waivered clinicians [physicians, nurse practitioners, and physician assistants], 1517 opioid treatment programs [providing methadone], and 5222 health care professionals who could prescribe extended-release naltrexone). Statistical analysis was performed from April 20, 2018, to May 8, 2019.

**Exposures:**

Demographic, workforce, lack of insurance, road density, urbanicity, opioid prescribing, and regional division county-level characteristics.

**Main Outcome and Measures:**

The outcome variable, “opioid high-risk county,” was a binary indicator of a high (above national) rate of opioid overdose mortality with a low (below national) rate of provider availability to deliver OUD medication. Spatial logistic regression models were used to determine associations with being an opioid high-risk county.

**Results:**

Of 3142 counties, 751 (23.9%) had high rates of opioid overdose mortality. A total of 1457 counties (46.4%), and 946 of 1328 rural counties (71.2%), lacked a publicly available OUD medication provider in 2017. In adjusted models, compared with the West North Central division, counties in the East North Central, Mountain, and South Atlantic divisions had increased odds of being opioid high-risk counties (East North Central: odds ratio [OR], 2.21; 95% CI, 1.19-4.12; Mountain: OR, 4.15; 95% CI, 1.34-12.89; and South Atlantic: OR, 2.99; 95% CI, 1.26-7.11). A 1% increase in unemployment was associated with increased odds (OR, 1.09; 95% CI, 1.03-1.15) of a county being an opioid high-risk county. Counties with an additional 10 primary care clinicians per 100 000 population had a reduced risk of being opioid high-risk counties (OR, 0.89; 95% CI, 0.85-0.93), as did counties that were micropolitan (vs metropolitan) (OR, 0.67; 95% CI, 0.50-0.90) and those that had an additional 1% of the population younger than 25 years (OR, 0.95; 95% CI, 0.92-0.98).

**Conclusions and Relevance:**

Counties with low availability of OUD medication providers and high rates of opioid overdose mortality were less likely to be micropolitan and have lower primary care clinician density, but were more likely to be in the East North Central, South Atlantic, or Mountain division and have higher rates of unemployment. Strategies to increase medication treatment must account for these factors.

## Key Points

**Question:**

What are the characteristics of US counties with high rates of opioid overdose mortality and low capacity to deliver medications for opioid use disorder?

**Findings:**

In this cross-sectional study of data from 3142 US counties, counties in the South Atlantic, Mountain, and East North Central divisions had more than twice the odds of being at high risk for opioid overdose mortality and lacking in capacity to deliver medications for opioid use disorder. Higher density of primary care clinicians, a younger population, micropolitan status, and lower rates of unemployment were associated with lower risk of opioid overdose and lower risk of lacking in capacity to deliver medications for opioid use disorder.

**Meaning:**

Strategies to address mortality from opioid overdose by increasing treatment for addiction should target urban counties in Appalachia, the Midwest, and the Mountain division and include efforts to increase primary care clinicians and employment opportunities.

## Teaser

This cross-sectional study examines characteristics of US counties with persistently high rates of opioid overdose mortality and low capacity to deliver medications for opioid use disorder.

## Introduction

Policy makers are striving to mitigate adverse consequences of the opioid crisis, which caused more than 130 deaths per day in 2017.^1^ The population with opioid use disorder (OUD)^2^ continues to grow, constituting between 2.1 million and 6 million individuals in 2017.^3,4,5^ With, at most, 20% to 40% of persons with OUD receiving treatment,^3,6,7^ policies that expand access to and delivery of evidence-based treatment are critical to reducing the risk of opioid overdose.^8,9,10^

Medications for OUD treatment (MOUDs) are the criterion standard for treating OUD. Three MOUDs—methadone hydrochloride, buprenorphine hydrochloride, and extended-release naltrexone hydrochloride—have all been shown in clinical trials to reduce opioid use and adverse health outcomes.^11,12,13,14,15,16,17,18,19,20,21,22,23^ Methadone treatment was associated with a 53% reduction and buprenorphine treatment was associated with a 37% reduction in all-cause mortality among patients with OUD compared with those receiving no MOUD in the 12 months after nonfatal overdose.^24^

However, evidence suggests that availability of MOUDs has been slow to expand and, in many cases, is not available.^24,25,26^ Only opioid treatment programs (OTPs), which are closely regulated at the federal and state levels, can deliver methadone^27^; this restriction has contributed to methadone’s short and relatively flat supply over time.^3,28^ Although some OTPs also supply buprenorphine products to treat OUD,^29,30^ a 2002 policy change that granted physicians in outpatient nonspecialty settings the authority to prescribe buprenorphine with training and a waiver issued by the federal Substance Abuse and Mental Health Services Agency (SAMHSA) led to greater availability of buprenorphine.^31,32^ Unlike methadone and buprenorphine, both opioid agonists, newer extended-release naltrexone is an opioid antagonist^33^ that can be prescribed by any licensed prescriber. However, patients must be opioid abstinent for at least 7 to 10 days prior to treatment with extended-release naltrexone, and data demonstrating this drug’s effectiveness in preventing overdose among those with a nonfatal overdose are lacking.^24^

Because evidence supports the use of MOUDs rather than other treatment modalities to reduce opioid overdose, there is a critical need to characterize areas where the need for, and availability of, MOUD treatment providers have a deleterious mismatch. Previous studies have examined availability of methadone and buprenorphine at the state level,^3,29,34^ the availability of buprenorphine alone,^29,34,35,36,37,38,39^ or the supply of substance use treatment facilities treating patients with MOUDs.^26,40^ Recent studies have examined the geospatial association between county-level buprenorphine and OTP supply and opioid overdose mortality,^41,42^ but none has characterized counties with a high overdose burden and low capacity to deliver MOUDs. Given the high level of intrastate variability in availability of MOUD providers^25,39^ and opioid overdose harms,^25^ as well as the importance of targeting resources to counties at highest risk of the mismatch between treatment and harms, we sought to fill that literature gap. We hypothesized that high rates of opioid analgesic prescribing, location in Appalachian regions, and low density of mental health care professionals and primary care clinicians would be associated with low supply of MOUD providers and high rates of opioid overdose mortality at the county level.

## Methods

### Study Populations and Data Sources

We analyzed characteristics associated with low availability of MOUD providers and high rates of opioid overdose mortality using a geospatial cross-sectional analysis design that combined county-level data from January 1, 2015, to December 31, 2017, from several sources with mortality data from the Centers for Disease Control and Prevention (CDC). Institutional review board approval was not required for this study that used all publicly available data, per the University of Michigan, Office of Research Operations Manual, Part 4.^43^ This study followed the Strengthening the Reporting of Observational Studies in Epidemiology (STROBE) guidelines for cross-sectional studies.^44^

#### MOUD Providers

The availability of OTPs and buprenorphine-waivered clinicians as of September 19, 2017, was determined from the publicly available SAMHSA provider locator websites.^28,45^ For OTPs, this constitutes a complete list of all 1517 OTP facilities licensed to provide methadone to treat OUD. For buprenorphine, this constitutes a complete list of 24 851 clinicians (physicians, nurse practitioners, and physician assistants) certified under federal law to prescribe buprenorphine through a waiver process^31,32^ and who agreed to be listed on the website for the purpose of being identified by patients seeking buprenorphine treatment. We obtained a comprehensive list from Alkermes Inc, the manufacturer of the extended-release naltrexone product Vivitrol, of the 5222 health care professionals listed on the company’s publicly available treatment locator searchable tool who were actively prescribing the medication as of November 30, 2017.^46^

For each of these 3 MOUDs, we geographically coded the addresses for all treatment providers. We then counted the number of providers per 100 000 county residents for each medication separately and across the 3 MOUDs aggregated. We used 2017 US American Community Survey (ACS) estimates of county populations as the denominator in these calculations.^47^

#### Opioid Overdose Deaths

**Figure 1.**
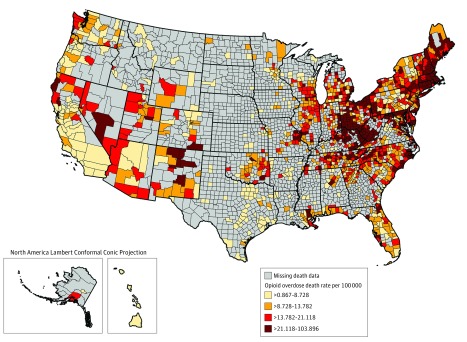
Opioid Overdose Death Rate per 100 000 People by US County, 2015-2017 Opioid-overdose deaths were classified using the *International Statistical Classification of Diseases and Related Health Problems, Tenth Revision* (*ICD-10*), based on the *ICD-10* underlying cause-of-death codes X40 toX 45 (unintentional), X60 to X65 (suicide), or Y10 to Y15 (undetermined intent). Among the deaths with drug overdose as the underlying cause, opioid overdose deaths were identified using the following *ICD-10* multiple cause-of-death codes: opium (T40.0), heroin (T40.1), natural and semisynthetic opioids (T40.2), methadone (T40.3), synthetic opioids excluding methadone (T40.4), or other and unspecific narcotics (T40.6).

We extracted county-level rates of opioid overdose mortality from January 1, 2015, to December 31, 2017, from the CDC WONDER (Wide-ranging Online Data for Epidemiologic Research) Multiple Cause of Death database.^48,49^ This database is based on US resident death certificates, which are coded into the National Vital Statistics System by states or the CDC’s National Center for Health Statistics. We searched for both intentional and unintentional underlying causes of death (*International Statistical Classification of Diseases and Related Health Problems, Tenth Revision* codes X40-X45, X60-X65, and Y10-Y15) where any type of opioid was coded for injury and poisoning (codes T40.0-T40.4 and T40.6). For the denominator, we used county populations aggregated for 2015-2017, to create county-level death rates per 100 000 residents per year. These death rates are depicted graphically in 1322 counties (1820 were suppressed) in Figure 1.

#### Opioid High-Risk Counties

We used the national opioid overdose mortality rate of 12.5 per 100 000 population from 2015-2017 as a threshold to divide counties with high and low opioid overdose death rates. We took the county MOUD provider availability rates across all 3 medication types (OTPs, buprenorphine-waivered clinicians, and extended-release naltrexone prescribers) and used the national rate of 9.7 providers per 100 000 residents as a threshold to divide counties into high and low MOUD provider capacity groups. We then defined *opioid high-risk counties* as counties with a low capacity for MOUD providers and a high rate of opioid overdose mortality. We analogously created county-level risk indicators for each MOUD separately (eAppendix in the Supplement).

#### Covariates

The county-level risk indicators consisted of demographic characteristics, density of primary care physicians (PCPs) and mental health clinicians, proportion uninsured, road density, urbanicity, opioid prescription rate, percentage voting democratic in the 2016 presidential election, and geographic regional division. We also conducted a sensitivity analysis that excluded clinician (PCP and mental health clinician) density, given that this factor is partially encompassed in the opioid high-risk county measure. County-level demographic characteristics—including age, race/ethnicity, unemployment, and educational level—were all taken from the ACS.^50^ County-level density of PCPs and mental health clinicians (counts per 100 000 population) were obtained from the Robert Wood Johnson Foundation County Health Rankings and Roadmaps 2016 data.^51^ County-level rates of uninsured individuals were drawn from the 2016 Small Area Health Insurance Estimates using the ACS.^52^ We obtained data on road mileage from the US Geological Survey National Geospatial Technical Operations Center’s 2006 National Transportation Data set and created a traversability variable by dividing road mileage by county land area from the ACS.^53^ County-level urbanicity was categorized as rural, micropolitan, and metropolitan using the National Center for Health Statistics Urban-Rural Classification Scheme for Counties.^54^ The number of opioid (analgesic) prescriptions dispensed per 100 persons in 2016 was drawn from the CDC.^55^ The political partisan measure was drawn from the Massachusetts Institute of Technology Election Data and Science Lab data.^56^ Geographic regional divisions were defined using the US Census Bureau’s 9-category categorization scheme (eFigure 1 in the Supplement).^57^

### Statistical Analysis

Statistical analysis was performed from April 20, 2018, to May 8, 2019. First, we described and mapped counties lacking any available MOUD provider (across all 3 medications) and by county-level urbanicity categories and the 9 census-based divisions. Next, we mapped the spatial distribution of the county MOUD provider density along a continuum and overlaid rates of opioid overdose mortality on top (eFigure 2 in the Supplement). Then we mapped the 4 county types (high and low MOUD provider rates by high and low rates of opioid overdose mortality) across the United States. We also created these spatial distribution maps disaggregated by MOUD type (eFigures 3-5 in the Supplement). Finally, we contrasted opioid high-risk counties with non–high-risk counties using 2-sample comparison tests on county-level demographics, density of PCPs and mental health care clinicians, county size and road density, urbanicity, opioid prescribing, percentage democratic vote, and regional division.

Our primary goal was to determine characteristics of opioid high-risk counties while addressing residual correlation arising from the spatially indexed nature of the outcome, and suppressed counties whose exact rate of opioid overdose mortality was unknown. To jointly address these issues we used a modification of logistic regression that models the residual spatial trends and incorporates weights based on the estimated likelihood that a suppressed county was actually an opioid high-risk county (eAppendix in the Supplement). We considered *P* < .05 to be statistically significant and used 2-sided tests. All models were adjusted for covariates enumerated above and fit using R, version 3.5.1 (R Project for Statistical Computing).

## Results

### Counties Lacking Any Publicly Available MOUD Treatment Provider

**Figure 2.**
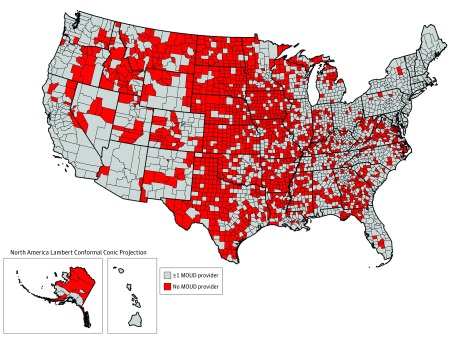
US Counties Lacking Any Publicly Available Medication for Opioid Use Disorder (MOUD) Provider, 2017 Medication for opioid use disorder providers are defined to include publicly listed opioid treatment programs, buprenorphine-waivered clinicians, and/or extended-release naltrexone–prescribing clinicians in late 2017.

eTable 1 in the Supplement shows the breakdown of 1457 of 3142 counties (46.4%) lacking any publicly listed MOUD treatment provider in late 2017, by geographic divisions and by urbanicity. Most counties in many divisions lacked any publicly available MOUD treatment provider, including in the West South Central (Arkansas, Louisiana, Oklahoma, and Texas) and West North Central (Iowa, Nebraska, Kansas, North Dakota, South Dakota, Minnesota, and Missouri) divisions. A total of 946 of 1328 rural counties (71.2%) lacked any publicly listed MOUD provider (Figure 2).

### Unadjusted Characteristics of Opioid High-Risk Counties

**Figure 3.**
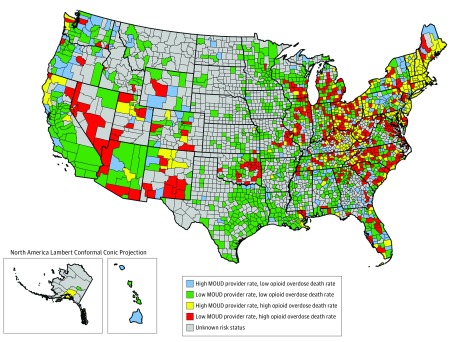
Opioid High-Risk Counties With Low Rates of Medication for Opioid Use Disorder (MOUD) Treatment Providers and High Rates of Opioid Overdose Death Low and high rates of MOUD providers defined as those below and greater than the national rate, respectively, in availability of 3 types of MOUD providers (publicly listed opioid treatment programs, buprenorphine-waivered clinicians, and extended-release naltrexone prescribers) in late 2017. Low and high rates of opioid overdose deaths defined as below and above the national rate of opioid overdose deaths, respectively, from 2015 to 2017.

Of 3142 US counties, 751 (23.9%) had high rates of opioid overdose mortality. Table 1 shows unadjusted contrasts between opioid high-risk counties and non–high-risk counties. Figure 3 depicts the spatial distribution of opioid high-risk counties and the other 3 categories designated as non–high-risk (low rates of MOUD providers and low rates of opioid overdose mortality; high rates of MOUD providers and low rates of opioid overdose mortality; and high rates of MOUD providers and high rates of opioid overdose mortality). We identified 412 opioid high-risk counties and 1485 non–high-risk counties. Owing to suppression of data on opioid overdose mortality, 1245 counties had inadequate data to be initially categorized by risk status for unadjusted results. In unadjusted analyses, opioid high-risk counties had greater proportions of the population that were white, unemployed, and lacking a high school education; these counties also had a lower proportion of the population younger than 25 years. Opioid high-risk counties also had lower concentrations of PCPs and mental health care clinicians per 100 000 persons, a higher rate of opioid prescriptions per 100 persons, and a lower percentage democratic vote in the 2016 presidential election. In addition, opioid high-risk counties were more likely than non–high-risk counties to be overrepresented in the South Atlantic and East North Central divisions.

**Table 1.** Characteristics of Opioid High-Risk Counties^a^

Characteristic	Mean (SD) Value			*P* Value^b^
	All Known Risk Counties (N = 1897)	Opioid High-Risk Counties (n = 412)	Non–High-Risk Counties (n = 1485)	
Male, %	49.7 (1.8)	49.7 (1.5)	49.7 (1.9)	.53
Clinician density per 100 000 population, No.				
Primary care clinicians^c^	62.9 (34.1)	51.4 (23.1)	66.2 (35.9)	<.001
Mental health clinicians^d^	158.2 (144.3)	125.3 (102.6)	167.4 (152.8)	<.001
Unemployed, %	7.5 (2.7)	7.8 (2.2)	7.3 (2.8)	<.001
Age, %				
<25 y	31.8 (4.5)	30.7 (3.7)	32.1 (4.6)	<.001
25-64 y	44.4 (3.1)	44.6 (2.7)	44.4 (3.2)	.18
White race, %	84.0 (15.1)	86.5 (13.0)	83.3 (15.6)	<.001
No high school or GED, %	13.6 (5.9)	13.9 (4.8)	13.5 (6.2)	.28
Uninsured, %	10.3 (4.5)	10.0 (3.9)	10.4 (4.7)	.17
Road length, mile^2^	4.8 (3.5)	4.8 (2.6)	4.8 (3.7)	.91
Opioid prescription rate per 100 population, No.^e^	84.6 (38.1)	88.6 (32.3)	83.5 (39.5)	.02
Democratic vote in 2016 presidential election, %^f^	34.9 (15.0)	31.4 (12.2)	35.8 (15.5)	<.001
Urbanicity, No. (%)^g^				
Rural	395 (20.8)	91 (22.1)	304 (20.5)	.21
Micropolitan	517 (27.3)	97 (23.5)	420 (28.3)	
Metropolitan	982 (51.8)	224 (54.4)	758 (51.0)	
Geographic division, No. (%)				
East North Central	326 (17.2)	116 (28.2)	210 (14.1)	<.001
Mid-Atlantic	146 (7.7)	23 (5.6)	123 (8.3)	
Mountain	145 (7.6)	31 (7.5)	114 (7.7)	
New England	66 (3.5)	5 (1.2)	61 (4.1)	
Pacific	129 (6.8)	8 (1.9)	121 (8.2)	
South Atlantic	415 (21.9)	141 (34.2)	274 (18.5)	
West North Central	192 (10.1)	13 (3.2)	179 (12.1)	
West South Central	225 (11.9)	20 (4.9)	205 (13.8)	
East South Central	253 (13.3)	55 (13.4)	198 (13.3)	

Abbreviation: GED, General Educational Development.

^a^ Opioid high-risk counties are defined as those with rates below the national rate in availability of 3 types of medication for opioid use disorder treatment providers combined in late 2017, and above the national opioid overdose death rate from 2015 to 2017.

^b^ *P* values for numerical variables were derived using the independent sample *t* tests. *P* values for categorical variables were derived Pearson χ^2^ 2-way tests for independent samples.

^c^ Primary care clinicians per 100 000 population. Missing 13 values among non–high-risk counties.

^d^ Mental health clinicians per 100 000 population. Missing 29 values (4 in opioid high-risk counties and 25 in non–high-risk counties).

^e^ Number of retail opioid prescriptions dispensed per 100 persons in 2016. Opioids include codeine phosphate, fentanyl citrate, hydrocodone bitartrate, hydromorphone hydrochloride, methadone hydrochloride, morphine sulfate, oxycodone hydrochloride, oxymorphone hydrochloride, propoxyphene hydrochloride, tapentadol hydrochloride, and tramadol hydrochloride, identified using the National Drug Code. Cough and cold formulations containing opioids, buprenorphine products typically used to treat opioid use disorder, and methadone dispensed through methadone maintenance treatment programs are excluded. Missing 11 (1 in an opioid high-risk county, and 10 in non–high-risk counties).

^f^ Missing 13 non–high-risk counties.

^g^ Urbanicity missing 3 non–high-risk counties.

Maps depicting the spatial distribution of opioid high-risk and non–high-risk counties with respect to each MOUD type independently are displayed in eFigures 3-5 in the Supplement. Generally, the geographic clusters of high-risk counties were similar across MOUD provider types, although risk distributions in the buprenorphine-waivered clinician maps (eFigure 3 in the Supplement) closely resemble the distributions shown in Figure 3 across all 3 medications. Slightly more high-risk counties emerged in the OTP and extended-release naltrexone clinician prescriber maps (eFigure 4 and eFigure 5 in the Supplement), albeit in similar regional divisions that house opioid high-risk counties in the buprenorphine and combined MOUD maps.

### Adjusted Characteristics of Opioid High-Risk Counties

Due to missing covariates (primarily counts of mental health clinicians and PCPs), high-risk probabilities could be estimated for only 831 of the 1245 initially uncategorized counties (eFigure 6 in the Supplement). Table 2 includes the spatial logistic regression results for characteristics associated with opioid high-risk counties, adjusted for county-level demographic, workforce, lack of insurance, road density, urbanicity, opioid prescribing, political partisanship, and regional division characteristics. Relative to the West North Central division, counties in the East North Central, Mountain, and South Atlantic divisions had increased odds of being opioid high-risk counties (East North Central: odds ratio [OR], 2.21; 95% CI, 1.19-4.12; Mountain: OR, 4.15; 95% CI, 1.34-12.89; and South Atlantic: OR, 2.99; 95% CI, 1.26-7.11). A 1% increase in unemployment was associated with an increased odds (OR, 1.09; 95% CI, 1.03-1.15) of a county being an opioid high-risk county. Counties that were micropolitan (vs metropolitan) had a reduced risk of being an opioid high-risk county (OR, 0.67; 95% CI, 0.50-0.90), as did counties with an additional 10 primary care clinicians per 100 000 population (OR, 0.89; 95% CI, 0.85-0.93) and those with an additional 1% of the population younger than 25 years (OR, 0.95; 95% CI, 0.92-0.98). An additional opioid prescription per 100 persons also was associated with marginally increased odds of opioid high-risk status (OR, 1.04; 95% CI, 1.00-1.07). In sensitivity results that excluded PCP and mental health clinician risk indicators, other risk indicators were substantively similar as in the main results. Opioid prescriptions were no longer a significant risk indicator, an additional percentage democratic vote corresponded to reduced odds (OR, 0.12; 95% CI, 0.03-0.44) of a county being an opioid high-risk county and an additional mile of road per square mile corresponded to reduced odds (OR, 0.95; 95% CI, 0.90-1.00) of a county being an opioid high-risk county (eTable 2 in the Supplement).

**Table 2.** Factors Associated With Opioid High-Risk Counties^a^^,^^b^

Factor	Odds Ratio (95% CI)	*P* Value
% Male	0.95 (0.89-1.02)	.15
Provider density per 100 000 population		
10 Primary care clinicians	0.89 (0.85-0.93)	<.001
10 Mental health clinicians	1.00 (0.99-1.01)	.78
% Unemployed	1.09 (1.03-1.15)	.001
% With no high school education or GED	0.95 (0.93-0.98)	.003
% Age		
<25 y	0.95 (0.92-0.98)	<.001
25-64 y	1.01 (0.96-1.05)	.76
>64 y	1 [Reference]	NA
% White race	1.00 (0.99-1.02)	.76
Road length, mile^2^	0.96 (0.91-1.01)	.14
% Uninsured	0.99 (0.95-1.04)	.70
Opioid prescription rate per 100 population	1.04 (1.00-1.07)	.02
% Democratic vote in 2016 presidential election	0.24 (0.05-1.05)	.06
Urbanicity		
Metropolitan	1 [Reference]	NA
Micropolitan	0.67 (0.50-0.90)	.009
Rural	0.85 (0.64-1.14)	.28
Regional division		
East North Central	2.21 (1.19-4.12)	.01
East South Central	1.72 (0.83-3.55)	.14
Mid-Atlantic	0.70 (0.25-1.99)	.50
Mountain	4.15 (1.34-12.89)	.01
New England	0.38 (0.07-2.10)	.27
Pacific	0.85 (0.15-4.93)	.86
South Atlantic	2.99 (1.26-7.11)	.01
West South Central	1.27 (0.62-2.59)	.51
West North Central	1 [Reference]	NA

Abbreviations: GED, General Educational Development; MH, mental health; NA, not applicable; PCP, primary care clinicians.

^a^ Opioid high-risk counties are those defined as those with rates below the national rate in public availability of 3 types of medication for opioid use disorder providers combined in late 2017 and above the national opioid overdose death rate from 2015 to 2017.

^b^ Models estimated using information from 2675 counties.

Adjusted regression results specific to each MOUD provider type are presented in eTables 3-5 in the Supplement. The factors for high-risk counties with respect to buprenorphine-waivered clinician availability are similar to those presented in Table 2. For low OTP provider availability paired with high rates of opioid overdose mortality, the East North Central division was no longer associated with greater risk and New England and the Mid-Atlantic divisions were associated with reduced risk. For the extended-release naltrexone models, the East South Central division had greater risk, whereas percentage democratic vote was associated with reduced risk.

## Discussion

We analyzed characteristics of counties that exhibited both historically high rates of opioid overdose mortality and low MOUD provider availability as of late 2017. Robust MOUD delivered via qualified providers in counties exhibiting these characteristics could realistically reduce opioid overdose deaths in vulnerable populations by as much as 40% to 60%.^24^ In terms of MOUD provider availability alone, we found that 46.4% of all counties and 71.2% of rural counties still lacked an OTP, buprenorphine-waivered clinician, or extended-release naltrexone–prescribing clinician identifiable through public listings. When we also considered treatment need, opioid high-risk counties tended to be overrepresented in the East North Central, South Atlantic, and Mountain divisions and had higher unemployment; micropolitan status was associated with lower risk of being an opioid high-risk county.

Prior work has examined the availability of MOUDs in various ways. Several studies have demonstrated that at the national and state levels, OTP availability has remained steady from 2003 to 2012.^3,37^ At the same time, numbers of Drug Addiction Treatment Act of 2000–waivered physicians capable of providing buprenorphine have significantly increased from 2003 to 2016.^3,34,37,39^ Increasing numbers of buprenorphine-waivered physicians are associated with higher rates of opioid overdose at the state level, suggesting that perhaps supply has been responsive to demand specific to this medication.^29,34^ Other state characteristics positively associated with rates of buprenorphine-waivered physicians in the literature include being in the Northeast region, the proportion of the population covered by Medicaid, the supply of OTPs, and the supply of substance use disorder treatment programs.^29^ Although shortages of buprenorphine-waivered clinicians have decreased over time, rurality is a persistent risk factor of shortage areas.^35,37,39^ In terms of specialty treatment facilities, past research has found shortages in the Southeast, Southwest, and Northeast regions.^26,40^ Geospatial analyses have found the greatest mismatch between OUD treatment programs and opioid overdose mortality in counties in Ohio, the District of Columbia, and West Virginia, and limited buprenorphine provider access relative to opioid overdose mortality throughout much of the Midwest and South.^41,42^

To our knowledge, this study is the first to present a picture of OUD treatment capacity across all 3 MOUDs and to compare this availability with recent historical need at the county level. These geospatial results indicate the specific types of counties where resources should be targeted to have greatest potential of increasing treatment and reducing overdose mortality. Given the characteristics of opioid high-risk counties that emerged from our main results, MOUD provider resources should be targeted to nonmicropolitan areas and in the East North Central (eg, Ohio, Michigan, Indiana, and Illinois), South Atlantic (eg, Virginia, West Virginia, the District of Columbia, Maryland, and Florida), and Mountain (eg, New Mexico, Arizona, Utah, and Nevada) divisions. Strategies to increase numbers of PCPs and other clinicians capable of and willing to provide MOUDs in these areas may be protective against a county persistently being high risk.^8^ Other innovative strategies to overcome workforce and geographic barriers—such as telemedicine, engagement of nonphysician prescribers in treatment, addressing stigma, providing peer-to-peer clinician support as in the Project ECHO (Extension for Community Healthcare Outcomes) model,^58^ providing hub and spoke models of OUD treatment along the continuum of care, expanding Medicaid to address health care access among low-income and unemployed individuals, and dispelling myths—are also likely needed.^8,59,60,61^

Recognizing that treatment with various MOUDs must be personalized based on patient factors, we also disaggregated county risk status by type of MOUD. Risk factors for buprenorphine were largely similar to those in analyses across all 3 MOUDS, suggesting that buprenorphine—the most available MOUD by far—was driving many of our main findings. Risk factors associated with buprenorphine were also consistent with prior studies of buprenorphine; namely, those that show risks of a shortage of buprenorphine to be associated with areas outside the northeast, particularly in the southeast.^29,34,37^ Our results differ in that they do not identify rurality as a risk factor, likely because of the more recent time frame of our mortality data, during which deaths from overdose of illicit opioids in urban settings were prevalent.

In the OTP model, greater availability of OTPs in the mid-Atlantic and New England divisions made counties in these regions less likely to be categorized as opioid high-risk counties, despite their high concentration of opioid overdose deaths from 2015 to 2017 (Figure 2). The New England and the mid-Atlantic divisions had higher availability of treatment providers across all 3 MOUDs, and some areas within them (eg, Massachusetts) have exhibited declines in rates of opioid overdose mortality starting in 2017.^62^ For high-risk status associated with extended-release naltrexone, the East South Central division (eg, Kentucky and Tennessee) served as an additional risk factor.

Disaggregation by medication type can help to inform intervention strategies. For example, areas with greater OTP capacity may be good candidates to employ hub and spoke models. Areas without OTP capacity (eg, the Mountain division) might instead consider enhanced telemedicine. According to all models, treatment options should be targeted in counties with high rates of unemployment, and potentially paired with initiatives to help individuals with OUD or in recovery to find employment. The “deaths of despair” hypothesis advanced by Case and Deaton,^63^ suggesting that structural determinants are key to opioid-associated harms, supports such approaches.^64^

### Limitations

Our study has several limitations. First, we used publicly available treatment locator data to assess the availability of MOUD providers from a patient’s or typical clinician’s viewpoint when identifying treatment options. Some buprenorphine-waivered clinicians do not consent to being on the buprenorphine list, making our shortage statistics overestimated,^39^ and we could be missing some MOUD providers not otherwise located by SAMHSA or Alkermes Inc. Alternatively, some buprenorphine-waivered providers who agree to be included on the buprenorphine list do not actively prescribe buprenorphine, potentially making our shortage statistics underestimated. Second, the Alkermes Inc list includes clinicians actively prescribing extended-release naltrexone, but does not differentiate whether this prescribing is for OUD or alcohol use disorder. Third, MOUD provider lists indicate availability, rather than volume of actual patient treatment. Most clinicians who prescribe buprenorphine prescribe the medication to a median monthly panel of 13 patients, many times even less^65,66^; thus, this measure may be an optimistic view of availability.

Fourth, our outcome variable of risk was subjectively determined, and treatment shortage or high-risk areas could be otherwise defined. Findings in studies specific to buprenorphine and OTP treatment that have used alternative definitions of this concept have been reasonably consistent with ours.^29,34,40,41,42^ Moreover, there could be some lag time in treatment availability responsive to overdose mortality, so the counties defined as opioid high-risk counties may no longer be high-risk in real time. Fifth, county-level opioid overdose death reporting drawn from national vital statistics data may include some measurement error, likely undercounting, that could be differential across counties.^67,68^ However, these counts are more reliable to infer causes of death than are other sources (eg, toxicology reports), are the best source of comparative death data available, have been previously analyzed at the county level, and are subject to analogous measurement error as occurs at the state level.^69,70,71^ Moreover, our outcome measure compared opioid overdose mortality with MOUD provider availability along continuums, rather than in absolute terms, which may have reduced the potential for biased conclusions. Sixth, we included many county-level covariates in our models, but it is possible there were unmeasured confounders not easily measured at the county level (eg, local county drug enforcement) that could be associated with opioid high-risk status. Seventh, we estimated high opioid overdose mortality probabilities in counties in which these counts were suppressed to add information to our adjusted models. Our findings were generally consistent between adjusted and unadjusted models, lending confidence that our weighting techniques were reasonable. Although we were able to impute information for most initially uncharacterized counties (831 of 1245 [66.7%]), adjusted results could have been biased by the omission of data from 414 predominantly rural counties.

## Conclusions

Policy makers in the United States increasingly recognize the inadequacy of MOUD treatment availability and its potential to significantly reduce overdose mortality, as evidenced by congressional activity emphasizing and substantial resources allocated for addiction treatment.^72,73^ This study provides new information to assist in identifying opioid high-risk counties and developing strategies to target resources. For instance, through the SAMHSA Substance Abuse Prevention and Treatment Block Grants to states and SUPPORT (Substance Use-Disorder Prevention that Promotes Opioid Recovery and Treatment) for Patients and Communities Act treatment augmentation provisions, prioritizing fund allocation and clinician workforce augmentation efforts around MOUD in nonmicropolitan counties, including in many Appalachian and Mountain regions, could be particularly effective in reducing opioid-related risks. In addition, focusing MOUD augmentation efforts in areas with fewer PCPs and higher unemployment rates would likely be an efficient use of resources to mitigate opioid harms. Although overall buprenorphine-waivered clinicians and funds for OUD treatment to states have increased in recent years, to have the largest effect on the opioid crisis these resources need to be funneled to local county areas with the greatest unmet need, together with new models of care to reach people with OUD.

## Notes

Supplement. **eAppendix.** Methods **eFigure 1.** U.S. Census Regional Divisions **eFigure 2.** County-Level Distribution of Medication for Opioid Use Disorder Treatment Provider Availability and Opioid Overdose Death Rates **eFigure 3.** U.S. Counties With Low Buprenorphine-Waivered Clinician Availability and High Opioid Overdose Death Rates **eFigure 4.** U.S. Counties With Low Opioid Treatment Program Availability and High Opioid Overdose Death Rates **eFigure 5.** U.S. Counties With Low Extended-Release Naltrexone Prescribing Clinician Availability and High Opioid Overdose Death Rates **eFigure 6.** Estimated Opioid High-Risk County Probabilities Among Suppressed Counties (N=831) **eTable 1.** Characteristics of U.S. Counties Lacking Any Available Medication for Opioid Use Disorder Treatment Provider, 2017 **eTable 2.** Characteristics of Opioid High-Risk Counties (Sensitivity) **eTable 3.** County Factors Associated With Low Buprenorphine-Waivered Clinician Availability and High Opioid Overdose Death Rates **eTable 4.** County Factors Associated With Low Opioid Treatment Program Availability and High Opioid Overdose Death Rates **eTable 5.** Factors Associated With Low Extended-Release Naltrexone Prescribing Clinician Availability and High Opioid Overdose Death Rates
