# Supplementary material for: Characteristics of US Counties With High Opioid Overdose Mortality and Low Capacity to Deliver Medications for Opioid Use Disorder
Source: JAMA Netw Open. 2019 Jun 28;2(6):e196373. doi: 10.1001/jamanetworkopen.2019.6373 (PMC6604101; doi:10.1001/jamanetworkopen.2019.6373)
Supplement: Supplement. — eAppendix. Methods eFigure 1. U.S. Census Regional Divisions eFigure 2. County-Level Distribution of Medication for Opioid Use Disorder Treatment Provider Availability and Opioid Overdose Death Rates eFigure 3. U.S. Counties With Low Buprenorphine-Waivered Clinician Availability and High Opioid Overdose Death Rates eFigure 4. U.S. Counties With Low Opioid Treatment Program Availability and High Opioid Overdose Death Rates eFigure 5. U.S. Counties With Low Extended-Release Naltrexone Prescribing Clinician Availability and High Opioid Overdose Death Rates eFigure 6. Estimated Opioid High-Risk County Probabilities Among Suppressed Counties (N=831) eTable 1. Characteristics of U.S. Counties Lacking Any Available Medication for Opioid Use Disorder Treatment Provider, 2017 eTable 2. Characteristics of Opioid High-Risk Counties (Sensitivity) eTable 3. County Factors Associated With Low Buprenorphine-Waivered Clinician Availability and High Opioid Overdose Death Rates eTable 4. County Factors Associated With Low Opioid Treatment Program Availability and High Opioid Overdose Death Rates eTable 5. Factors Associated With Low Extended-Release Naltrexone Prescribing Clinician Availability and High Opioid Overdose Death Rates [file jamanetwopen-2-e196373-s001.pdf]

Supplementary Online Content

Haffajee RL, Lin LA, Bohnert ASB, Goldstick JE. Characteristics of US counties with high opioid overdose mortality and low capacity to deliver medications for opioid use disorder. *JAMA Netw Open.* 2019;2(6):e196373. doi:10.1001/jamanetworkopen.2019.6373

eAppendix. Methods
eFigure 1. U.S. Census Regional Divisions
eFigure 2. County-Level Distribution of Medication for Opioid Use Disorder Treatment Provider Availability and Opioid Overdose Death Rates
eFigure 3. U.S. Counties With Low Buprenorphine-Waivered Clinician Availability and High Opioid Overdose Death Rates
eFigure 4. U.S. Counties With Low Opioid Treatment Program Availability and High Opioid Overdose Death Rates
eFigure 5. U.S. Counties With Low Extended-Release Naltrexone Prescribing Clinician Availability and High Opioid Overdose Death Rates
eFigure 6. Estimated Opioid High-Risk County Probabilities Among Suppressed Counties (N=831)
eTable 1. Characteristics of U.S. Counties Lacking Any Available Medication for Opioid Use Disorder Treatment Provider, 2017
eTable 2. Characteristics of Opioid High-Risk Counties (Sensitivity)
eTable 3. County Factors Associated With Low Buprenorphine-Waivered Clinician Availability and High Opioid Overdose Death Rates
eTable 4. County Factors Associated With Low Opioid Treatment Program Availability and High Opioid Overdose Death Rates
eTable 5. Factors Associated With Low Extended-Release Naltrexone Prescribing Clinician Availability and High Opioid Overdose Death Rates

This supplementary material has been provided by the authors to give readers additional information about their work.

© 2019 Haffajee RL et al. *JAMA Network Open*.

eAppendix. Methods

Before creating our opioid high-risk county variable and maps and for data transparency, we mapped the distribution of MOUD providers (zero and quartiles not including 0) and overlaid the quartile distribution of opioid-overdose deaths (eFigure 2).

In our adjusted models that identify the characteristics of opioid high-risk counties, we addressed two issues in our modeling: (a) residual correlation arising from the spatially-indexed nature of the outcome, which is important to protect the veracity of our inference that would be damaged by unmodeled spatial (e.g., between-county) dependence; and (b) suppressed counties whose exact opioid-overdose death rate was unknown, which could generate bias if those counties are inherently different from unsuppressed counties. We used a modification of logistic regression that models the residual spatial trends, and incorporates weights based on the estimated likelihood that a suppressed county was actually high-risk (1):

$$\log\left(\frac{P(Y_i = 1\,|\boldsymbol{X_i})}{P(Y_i = 0\,|\boldsymbol{X_i})}\right) = \boldsymbol{X_i}\boldsymbol{\beta} + f(\mathbf{s}_i). \quad (1)$$

The function $f(\mathbf{s}_i)$ is a non-parametrically estimated thin-plate spline function of the spatial coordinates of the county i centroid, $\mathbf{s}_i$, and models any residual spatial trend. This approach removes residual spatial dependency without requiring prior specification of the dependency structure, and while avoiding computational difficulties arising from large spatial adjacency matrices. Within this model we derived adjusted covariate effect estimates. These models were fit using the R package *mgcv*.

To address death count suppression, we followed a three-step process: (i) determine the number of opioid-overdose deaths required for a given county, based on its population size, to have a high opioid-overdose death rate; (ii) estimate covariate effects on opioid-overdose death rates using censored Poisson regression, treating suppressed counties as having counts between 0 and 9; and (iii) use the predictions from the fitted model to determine each suppressed county's probability of exceeding the threshold determined in part (i). The model fit in step (ii) included an offset for the log population size, and all of the covariates included in the main model. When the MOUD provider rate is high (≥ the median), or when the maximum suppressed overdose count (9) still results in a low overdose rate, a suppressed county is known not to be an opioid high-risk county. We incorporated those predicted probabilities into the model (1) by weighting cases; for example, for a case with 60% predicted probability of being an opioid high-risk county, two rows were entered into the data set—one with $Y_i = 1$ and one with $Y_i = 0$, weighted by 0.6 and 0.4, respectively. These censored Poisson models were fit using custom R code.

Among the 1245 counties whose risk status was unknown, opioid high-risk county probabilities could be estimated among 831; the other 414 had missing covariate values, mostly due to the primary care clinician and mental health care clinician variables. eFigure 6 shows a histogram of the estimated opioid high-risk county probabilities for those 831 counties. The counties with probabilities far from 50% are those that impact effective sample size the most; 534 (65.3%) had >75% chance of being a non-high-risk county; another 10 (1.2%) had >75% of being an opioid high-risk county.

© 2019 Haffajee RL et al. *JAMA Network Open*.

eFigure 1. U.S. Census Regional Divisions[a]

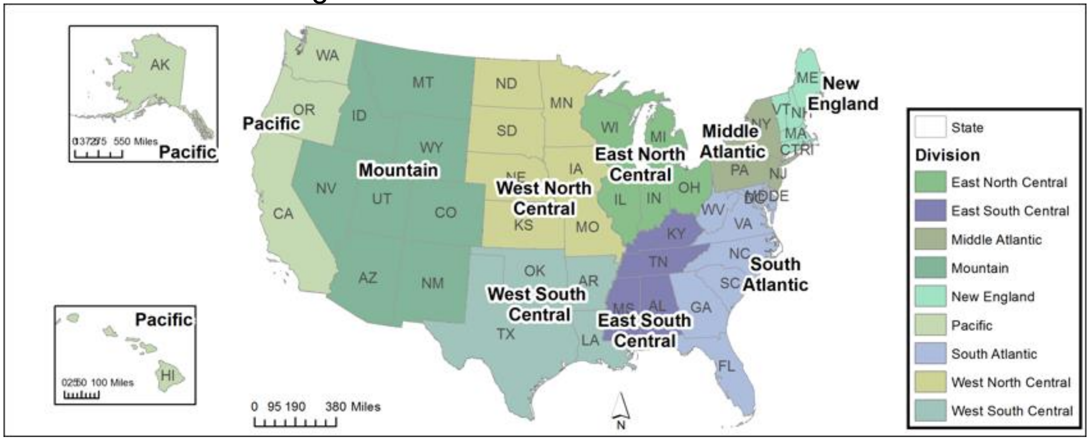

Notes:
[a] The U.S. Census Bureau defines nine census divisions set forth in this map.[57]

© 2019 Haffajee RL et al. *JAMA Network Open*.

eFigure 2. County-Level Distribution of Medication for Opioid Use Disorder (MOUD)[a] Provider Availability[b] and Opioid Overdose Death Rates[c]

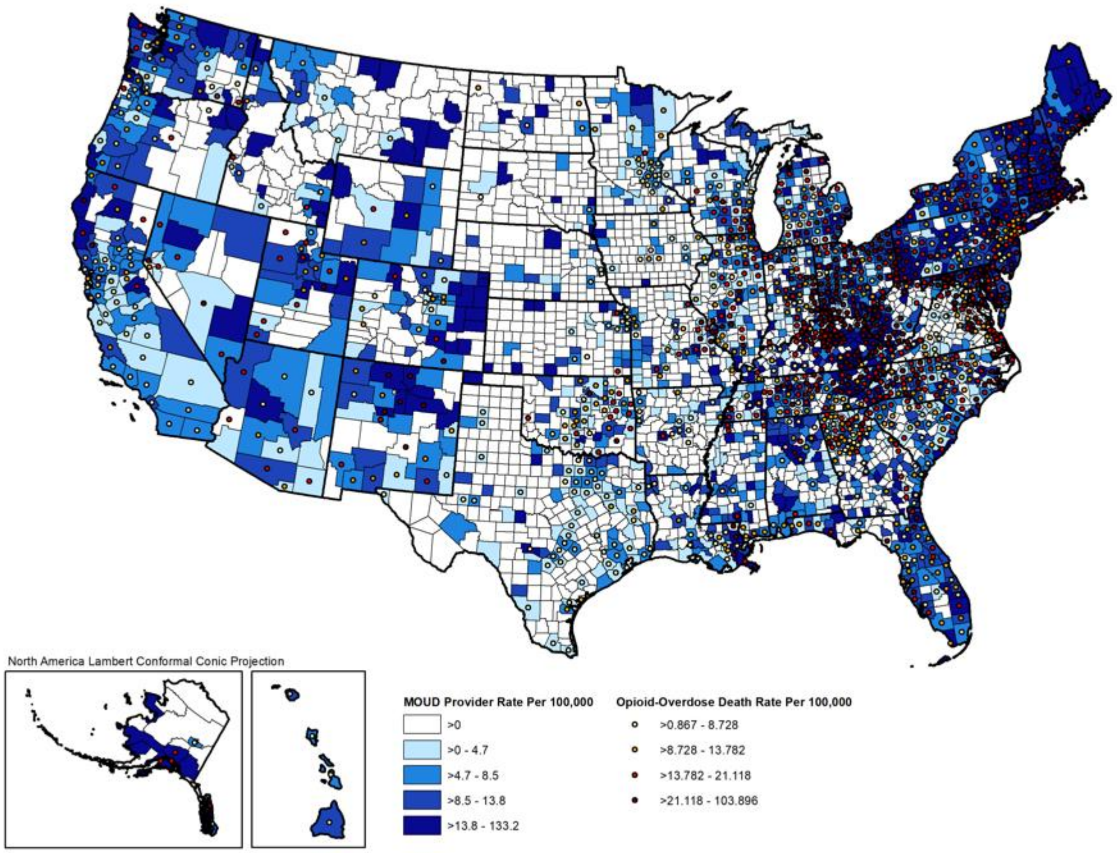

Notes:

[a] MOUD, Medication for opioid use disorder.

[b] MOUD provider availability calculated using treatment locator information for all three MOUD medications (opioid treatment programs, buprenorphine-waivered clinicians, and extended-release naltrexone prescribers) in late 2017.

[c] Opioid-overdose deaths in 2015-2017 were classified using the International Classification of Disease, Tenth Revision (ICD-10), based on the ICD-10 underlying cause-of-death codes X40–X45 (unintentional), X60–X65 (suicide), or Y10–Y15 (undetermined intent). Among the deaths with drug overdose as the underlying cause, opioid-overdose deaths were identified using the following ICD-10 multiple cause-of-death codes: opium (T40.0), heroin (T40.1), natural and semi-synthetic opioids (T40.2), methadone (T40.3), synthetic opioids excluding methadone (T40.4), or other and unspecific narcotics (T40.6).

© 2019 Haffajee RL et al. *JAMA Network Open*.

eFigure 3. U.S. Counties With Low Buprenorphine-Waivered Clinician Availability and High Opioid Overdose Death Rates[a]

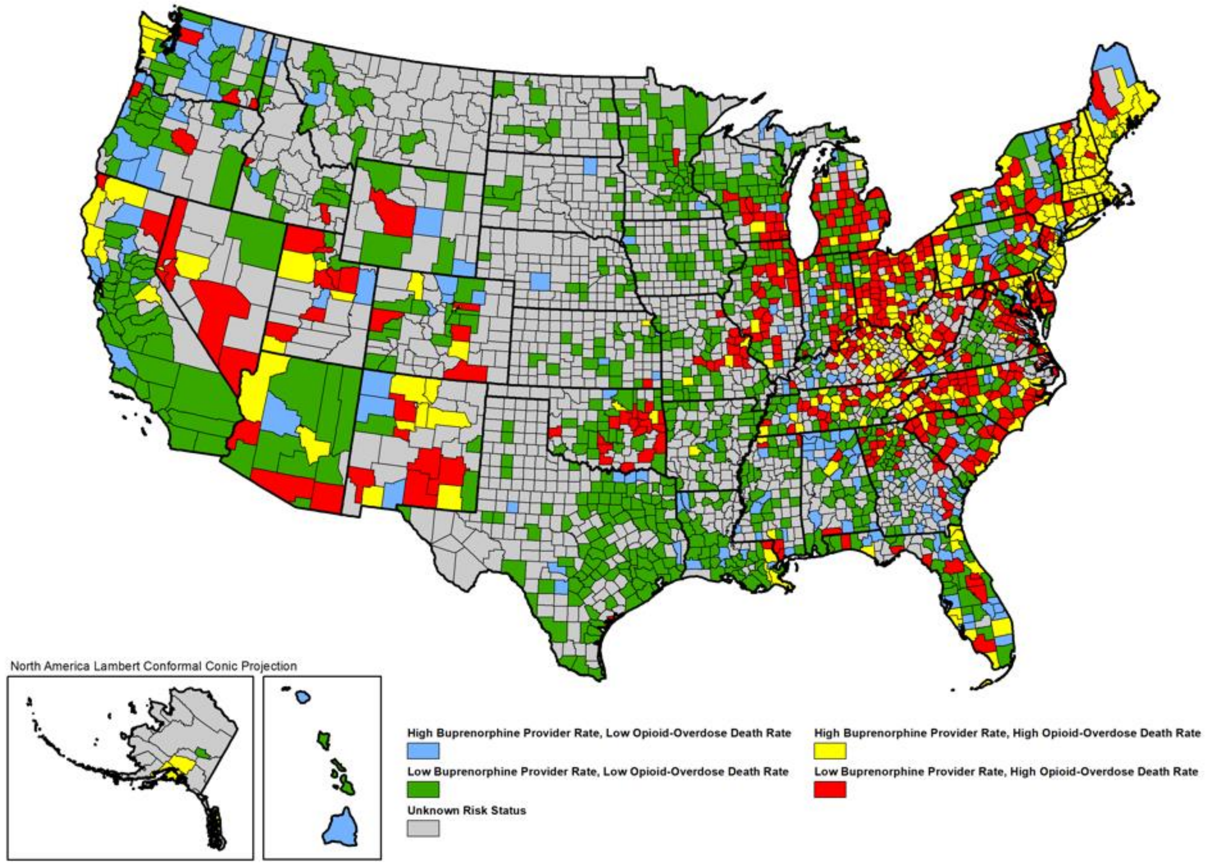

Notes:
[a] High-risk counties in red are those with rates: (1) below the national rate in availability of publicly-listed buprenorphine-waivered clinicians, 2017, and (2) above the national opioid-overdose deaths, 2015-2017.

© 2019 Haffajee RL et al. *JAMA Network Open*.

eFigure 4. U.S. Counties With Low Opioid Treatment Program Availability and High Opioid Overdose Death Rates[a]

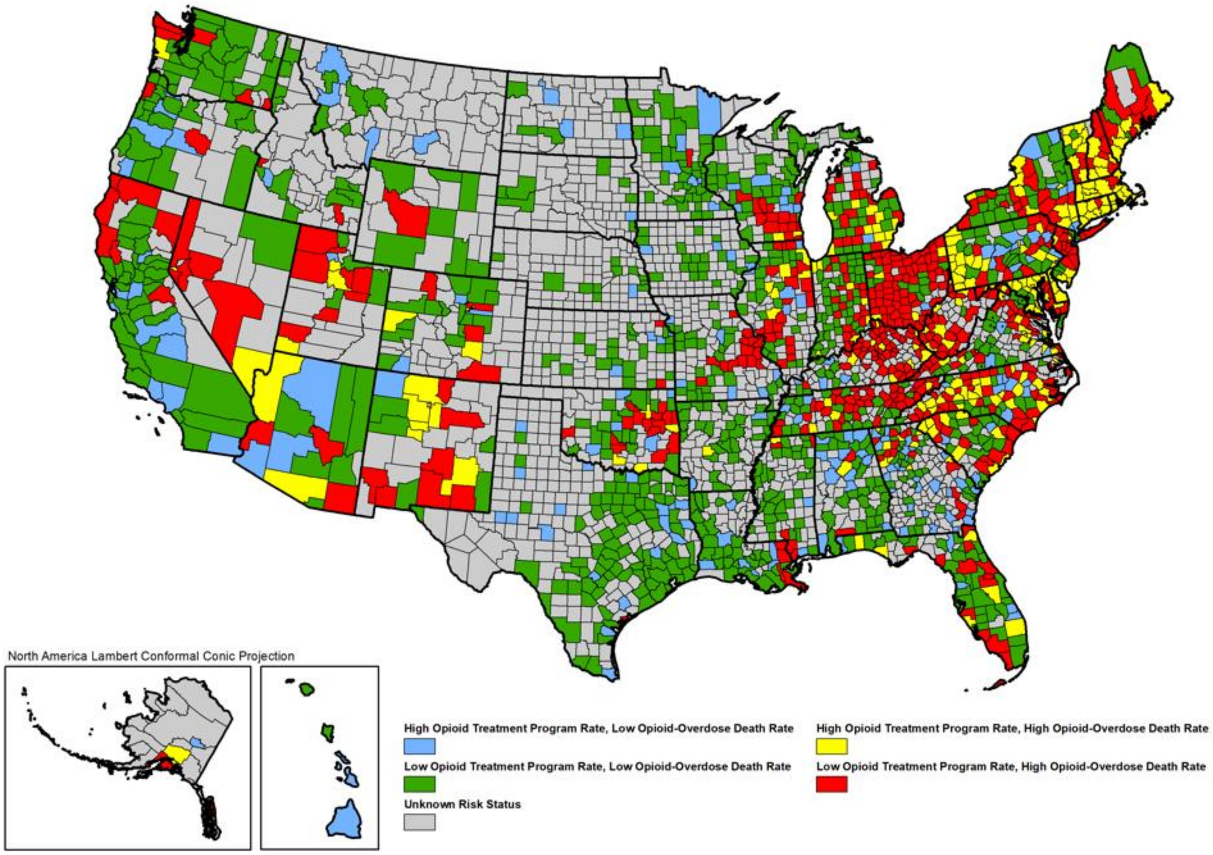

Notes:
[a] High-risk counties in red are those with rates: (1) below the national rate in availability of publicly-listed opioid treatment programs, 2017, and (2) above the national opioid-overdose deaths, 2015-2017.

© 2019 Haffajee RL et al. *JAMA Network Open*.

eFigure 5. U.S. Counties With Low Extended-Release Naltrexone Prescribing Clinician Availability and High Opioid Overdose Death Rates[a]

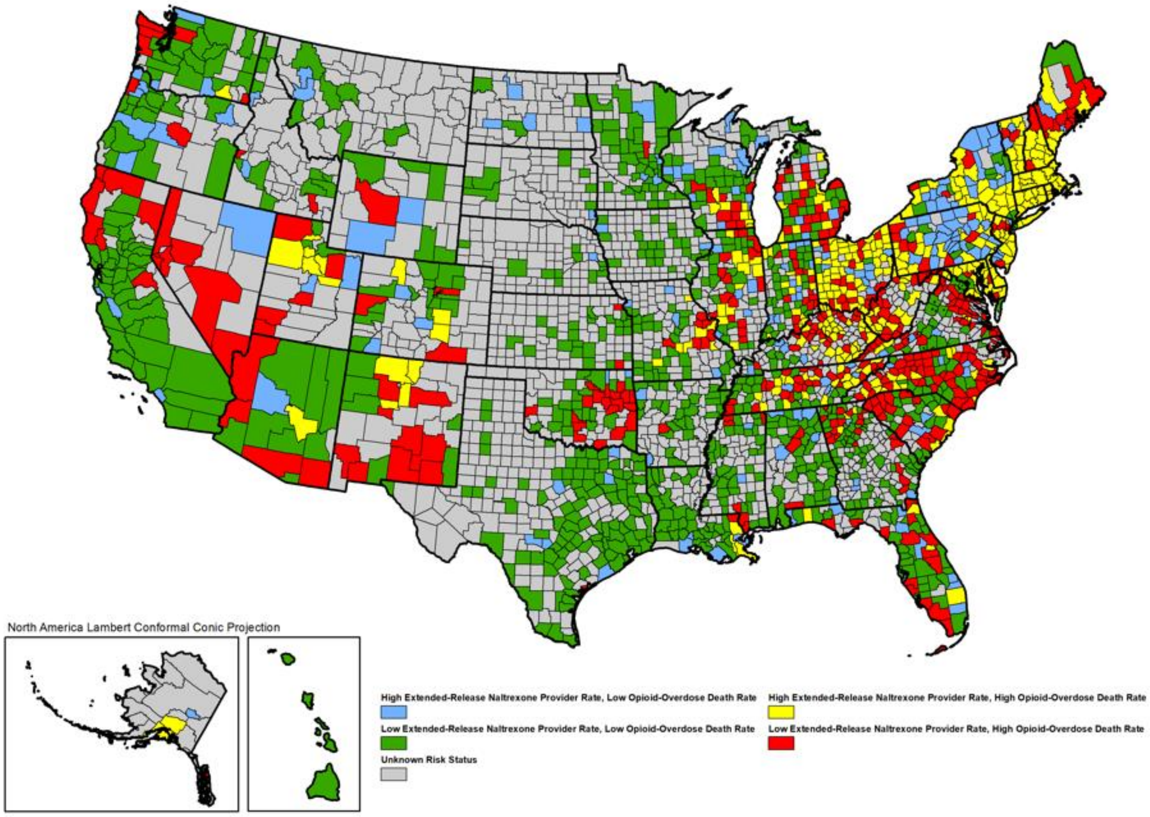

Notes:
[a] High-risk counties in red are those with rates: (1) below the national rate in availability of publicly-listed extended-release naltrexone prescribing clinician, 2017, and (2) above the national opioid-overdose deaths, 2015-2017.

© 2019 Haffajee RL et al. *JAMA Network Open*.

eFigure 6. Estimated Opioid High-Risk County Probabilities Among Suppressed Counties[a] (N=831)

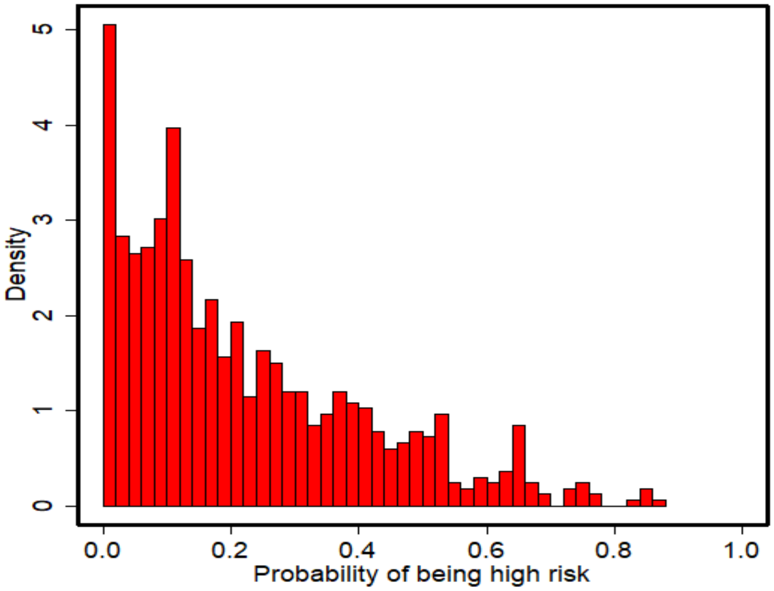

Notes:
[a] Y-axis reflects densities associated with each binning of the high-risk probabilities. We could not estimate high-risk probabilities among N=414 suppressed counties due to missing covariate values (primarily among the primary care clinician and mental health care clinician variables).

© 2019 Haffajee RL et al. *JAMA Network Open*.

eTable 1. Characteristics of U.S. Counties Lacking Any Available Medication for Opioid Use Disorder Treatment Provider, 2017

	**Counties with No Available MOUD[a] Provider[b]**
Division: N (% in Division)	
East South Central	143 (39.3%)
Pacific	41 (24.6%)
Mountain	134 (47.7%)
West South Central	298 (63.4%)
New England	1 (1.5%)
South Atlantic	213 (36.2%)
East North Central	155 (35.5%)
West North Central	468 (75.7%)
Mid-Atlantic	4 (2.7%)
Urbanicity: N (% in Urbanicity Category)	
Rural	946 (71.2%)
Micropolitan	222 (34.6%)
Metro	285 (24.4%)
Total: N(% of all counties)	1457 (46.4%)

Notes:
[a] MOUD, medication for opioid use disorder
[b] MOUD provider defined to include publicly-listed opioid treatment programs, buprenorphine-waivered clinicians, and extended-release naltrexone prescribers in late 2017.

© 2019 Haffajee RL et al. *JAMA Network Open*.

eTable 2. Characteristics of Opioid High-Risk Counties[a] (Sensitivity)[b]

	**Odds Ratio**	**95% Confidence Interval**	**p-value**
% Male	0.96	(0.90, 1.02)	0.16
% Unemployed	1.10	(1.05, 1.15)	<0.001
% No High School Education/GED[c]	0.98	(0.96, 1.01)	0.22
Age (Reference: % Over 64)			
% Under 25	0.94	(0.92, 0.97)	< 0.001
% 25-64	1.00	(0.96, 1.04)	0.95
% White	1.00	(0.99, 1.01)	0.84
Road Length/Square Mile	0.95	(0.90, 1.00)	0.03
% Uninsured	0.97	(0.94, 1.01)	0.21
Opioid Prescription/100	1.01	(0.99, 1.04)	0.42
% Democratic Vote	0.12	(0.03, 0.44)	0.001
Urbanicity (Reference: Metropolitan)			
Micropolitan	0.64	(0.48, 0.85)	0.002
Rural	0.82	(0.62, 1.07)	0.15
Regional Division (Reference: West North Central)			
East North Central	2.32	(1.32, 4.09)	0.004
East South Central	1.59	(0.82, 3.09)	0.17
Mid-Atlantic	0.63	(0.24, 1.67)	0.35
Mountain	2.54	(1.03, 6.25)	0.04
New England	0.33	(0.07, 1.57)	0.16
Pacific	0.46	(0.10, 2.15)	0.33
South Atlantic	2.66	(1.19, 5.96)	0.02
West South Central	1.16	(0.61, 2.22)	0.65

Notes:
[a] Opioid high-risk counties are defined as those with rates: (1) below the national rate in availability of three types of medication for opioid use disorder treatment providers combined in late 2017, and (2) above the national opioid-overdose death rate from 2015-2017
[b] Models estimated using information from 2989 counties.
[c] GED, General Educational Development.

© 2019 Haffajee RL et al. *JAMA Network Open*.

eTable 3. County Factors Associated With Low Buprenorphine-Waivered Clinician Availability[a] and High Opioid Overdose Death Rates[b,c]

	Odds Ratio	95% Confidence Interval	p-value
% Male	0.95	(0.89, 1.02)	0.15
Clinician Density			
10 PCPs/100,000[d]	0.90	(0.86, 0.94)	<0.001
10 MH clinician/100,000[e]	1.00	(0.99, 1.01)	0.76
% Unemployed	1.07	(1.02, 1.13)	0.006
% No High School Education/GED[f]	0.97	(0.94, 1.00)	0.03
Age (Reference: % Over 64)			
% Under 25	0.96	(0.93, 0.99)	0.005
% 25-64	1.01	(0.96, 1.05)	0.82
% White	1.00	(0.99, 1.02)	0.90
Road Length/Square Mile	0.96	(0.91, 1.01)	0.10
% Uninsured	0.96	(0.92, 1.01)	0.10
Opioid Prescription/100	1.04	(1.01, 1.07)	0.02
% Democratic Vote	0.15	(0.03, 0.65)	0.01
Urbanicity (Reference: Metropolitan)			
Micropolitan	0.73	(0.54, 0.97)	0.03
Rural	0.86	(0.64, 1.15)	0.31
Regional Division (Reference: West North Central)			
East North Central	2.30	(1.28, 4.14)	0.005
East South Central	1.33	(0.67, 2.66)	0.41
Mid-Atlantic	0.47	(0.18, 1.26)	0.14
Mountain	3.86	(1.36, 10.9)	0.01
New England	0.22	(0.05, 1.05)	0.06
Pacific	1.02	(0.18, 5.60)	0.99
South Atlantic	2.30	(1.00, 5.27)	0.05
West South Central	1.32	(0.67, 2.58)	0.42

Notes:
[a] Counties with low buprenorphine-waivered clinician availability defined as those with rates below the national rate in public availability of buprenorphine-waivered clinicians in late 2017.
[b] Counties with high opioid-overdose deaths defined as those with rates above the national opioid-overdose death rate from 2015-2017.
[c] Models estimated using information from 2675 counties.
[d] PCP/100,000, primary care clinicians per 100,000 population.
[e] MH clinicians/100,000, mental health clinicians per 100,000 population.
[f] GED, General Educational Development.

© 2019 Haffajee RL et al. *JAMA Network Open*.

eTable 4. County Factors Associated With Low Opioid Treatment Program Availability[a] and High Opioid Overdose Death Rates[b,c]

	Odds Ratio	95% Confidence Interval	p-value
% Male	0.99	(0.92, 1.06)	0.75
Clinician Density			
10 PCPs/100,000[d]	0.94	(0.91, 0.98)	0.005
10 MH clinicians/100,000[e]	1.00	(0.99, 1.01)	0.66
% Unemployed	1.06	(1.01, 1.11)	0.03
% No High School Education/GED[f]	0.97	(0.94, 1.00)	0.03
Age (Reference: % Over 64)			
% Under 25	0.92	(0.90, 0.95)	<0.001
% 25-64	0.99	(0.94, 1.03)	0.52
% White	1.01	(0.99, 1.02)	0.46
Road Length/Square Mile	1.00	(0.95, 1.04)	0.89
% Uninsured	0.98	(0.94, 1.03)	0.40
Opioid Prescription/100	1.03	(1.00, 1.07)	0.03
% Democratic Vote	0.38	(0.09, 1.55)	0.18
Urbanicity (Reference: Metropolitan)			
Micropolitan	1.02	(0.77, 1.34)	0.91
Rural	1.12	(0.84, 1.49)	0.43
Regional Division (Reference: West North Central)			
East North Central	1.34	(0.69, 2.57)	0.39
East South Central	1.56	(0.73, 3.34)	0.25
Mid-Atlantic	0.21	(0.07, 0.61)	0.004
Mountain	5.13	(1.46, 17.99)	0.01
New England	0.10	(0.02, 0.47)	0.004
Pacific	1.58	(0.26, 9.44)	0.62
South Atlantic	0.63	(0.26, 1.55)	0.31
West South Central	1.43	(0.67, 3.06)	0.35

Notes:
[a] Counties with low opioid treatment program availability defined as those with rates below the national rate in availability of these types of providers as publicly-listed in late 2017.
[b] Counties with high opioid-overdose deaths defined as those with rates above the national opioid-overdose death rate from 2015-2017.
[c] Models estimated using information from 2675 counties.
[d] PCP/100,000, primary care clinicians per 100,000 population.
[e] MH clinicians/100,000, mental health clinicians per 100,000 population.
[f] GED, General Educational Development.

© 2019 Haffajee RL et al. *JAMA Network Open*.

eTable 5. Factors Associated With Low Extended-Release Naltrexone Prescribing Clinician Availability[a] and High Opioid Overdose Death Rates[b,c]

	Odds Ratio	95% Confidence Interval	p-value
% Male	0.96	(0.90, 1.03)	0.30
Provider Density			
10 PCPs/100,000[d]	0.93	(0.89, 0.97)	0.001
10 MH providers/100,000[e]	1.01	(1.00, 1.01)	0.21
% Unemployed	1.10	(1.05, 1.16)	<0.001
% No High School Education/GED[f]	0.97	(0.94, 1.00)	0.07
Age (Reference: % Over 64)			
% Under 25	0.93	(0.90, 0.96)	<0.001
% 25-64	1.01	(0.96, 1.05)	0.78
% White	1.00	(0.99, 1.02)	0.70
Road Length/Square Mile	1.01	(0.96, 1.05)	0.74
% Uninsured	1.01	(0.96, 1.05)	0.80
Opioid Prescription/100	1.03	(1.00, 1.06)	0.04
% Democratic Vote	0.21	(0.05, 0.90)	0.04
Urbanicity (Reference: Metropolitan)			
Micropolitan	0.67	(0.50, 0.91)	0.01
Rural	0.94	(0.70, 1.25)	0.65
Regional Division (Reference: West North Central)			
East North Central	2.04	(1.05, 4.00)	0.04
East South Central	3.27	(1.51, 7.06)	0.003
Mid-Atlantic	1.17	(0.37, 3.63)	0.79
Mountain	4.81	(1.50, 15.42)	0.008
New England	1.96	(0.37, 10.39)	0.43
Pacific	1.00	(0.18, 5.53)	0.99
South Atlantic	5.89	(2.36, 14.67)	<0.001
West South Central	1.75	(0.84, 3.67)	0.14

Notes:
[a] Counties with low extended-release naltrexone prescribing clinician availability defined as those with rates below the national rate in availability of these types of clinicians as publicly-listed in late 2017.
[b] Counties with high opioid-overdose deaths defined as those with rates above the national opioid-overdose death rate from 2015-2017.
[c] Models estimated using information from 2675 counties.
[d] PCP/100,000, primary care clinicians per 100,000 population.
[e] MH clinicians/100,000, mental health clinicians per 100,000 population.
[f] GED, General Educational Development.

© 2019 Haffajee RL et al. *JAMA Network Open*.
